# Effect of restrictive fluid resuscitation on the coagulation function and hemodynamic parameters in patients with hemorrhagic traumatic shock

**DOI:** 10.1016/j.clinsp.2023.100300

**Published:** 2023-11-04

**Authors:** Junfen Zheng, Jinqiang Zhu, Liexiang Cao, Meiping Dong, Yi Mao, Zhiwei Zhao, Yao Liu

**Affiliations:** Department of Emergency Center, The First People's Hospital of Wenling, Wenling, China

**Keywords:** Hemorrhagic traumatic shock, Restrictive fluid resuscitation, Blood coagulation, Incidence rate of complication, Cure rate

## Abstract

**Objectives:**

To investigate the changes in the coagulation function and hemodynamic parameters in patients with Hemorrhagic Traumatic Shock (HTS) after restrictive fluid resuscitation.

**Methods:**

A total of 139 patients with HTS admitted to our hospital were enrolled, among which 69 HTS patients were divided into the control group and the remaining 70 HTS patients as the observation group. Patients in the control group underwent regular fluid resuscitation, while those in the observation group underwent restrictive fluid resuscitation.

**Results:**

During treatment, 70 patients in the observation group had a lower bleeding amount, infusion amount, and blood transfusion volume than those in the control group (p *<* 0.05). After treatment, patients in the observation group had better hemodynamic parameters and blood coagulation than those in the control group (p *<* 0.05), and the incidence rate in the observation group was only 12.9%, which was significantly lower than 60.87% in the control group, while the cure rate in the observation group was 100%, which was significantly higher than that in the control group (p *<* 0.05).

**Conclusions:**

Restrictive fluid resuscitation could remarkably increase the cure rate and reduce the bleeding amount during HTS treatment, thereby benefiting the recovery of the patient's blood coagulation.

## Highlights

• It is able to as the balance point between the blood pressure and bleeding event.
• It can reduce the bleeding amount and infusion volume and increase the cure rate of patients.
• Restrictive fluid resuscitation has a promising value in the treatment of HTS, which is worthy of promotion in clinical practice.

## Introduction

Hemorrhagic Traumatic Shock (HTS) is a common and severe disease in the emergency department and is one of the most common causes of death.^1^ Current strategies for HTS treatment are designed to stop bleeding via the infusion of blood and fluid and restore the arterial transport of blood oxygen.^2^,^3^ Early hypoperfusion or shock has been shown to cause coagulation dysfunction in patients.^3^, ^4^, ^5^ Approximately 25% of patients with severe traumatic injury were admitted with coagulopathy.^3^,^5^,^6^ Hemorrhagic shock and coagulopathy on admission are independently associated with heavy transfusion and increased mortality. So far, rapid intravenous infusion of the crystalloid plasma substitute is considered the mainstream treatment strategy for HTS, which could restore arterial pressure and maintain pressure before bleeding.

HTS is one of the most severe emergency clinical conditions,^7^ which can cause serious complications. To mitigate such effects, damage control strategies have been proposed, including early control of bleeding and adequate fluid resuscitation. The purpose of antihypertensive resuscitation is to maintain systolic (or mean arterial) blood pressure to maintain organ perfusion.^7^,^8^ Fluid resuscitation is a common method for HTS treatment and has made great progress in recent years.^9^ The efficacy of different fluid resuscitation methods may differ for varying diseases and conditions. Recently, Safiejko et al. conducted a meta-analysis of fluid resuscitation with hypertonic saline and dextran, and as a result, it performed better for hypotensive, hemorrhagic shock patients than those who underwent regular fluid resuscitation.^10^ Besides, for patients with infectious shock, persistent hyperlactatemia is believed to be a signal of insufficient tissue perfusion, and current evidence shows that lactic acid-targeted liquid resuscitation works well for treating patients with infectious shock.^11^ Furthermore, to clarify the correlation between the volume of infused liquid and a poor prognosis, Safiejko et al. performed a meta-analysis including data from 28 randomized controlled trials involving 4503 patients and found that restrictive fluid resuscitation significantly reduced mortality in patients with hypovolemic shock.^12^

Therefore, a promising method of fluid resuscitation may be conducive to HTS treatment. In this study, the authors performed restrictive fluid resuscitation for the treatment of HTS by infusing 5% hydroxyethyl starch in chloride sodium into patients with HTS and sustained the mean arterial pressure between 61 and 70 mmHg, thereby uncovering the efficacy of restrictive fluid resuscitation for HTS and its effect on the prognosis of patients with HTS.

## Materials and methods

### Patients

A total of 139 patients with traumatic hemorrhage-induced shock treated at the Department of Emergency of the First People's Hospital of Wenling between September 2017 and November 2020 were enrolled, of which 69 patients with HTS were divided into the control group and the remaining patients as the observation group. Patients in the control group received regular fluid resuscitation, while those in the observation group underwent restrictive fluid resuscitation. In all the patients with HTS, the volume of infused fluid was less than 1000 mL, systolic pressure was lower than 90 mmHg, and pulse pressure was less than 20 mmHg. This study was approved by the Ethics Committee of the First People's Hospital of Wenling (protocol n° KY-2023-2003-01) and followed the CONSORT Statement rules. All study participants provided written informed consent prior to participating in the study.

### Treatment

Immediately after the HTS attack, patients were treated with regular first aid measures to maintain the opening of the airway and oxygen uptake, sustain the temperature, and perform peripherally inserted central catheter intubation, while two venous channels were established. Blood samples were then collected from all patients with HTS for routine blood analysis, arterial blood gas analysis, evaluation of hepatic and renal function, and blood coagulation while monitoring the circulation system, arterial blood gas, lactic acid, physical signs, and central venous pressure. Regular fluid resuscitation was performed through two infusion channels. Through one channel, 1000 mL crystalloid solution was infused rapidly within 20 min, followed by the infusion of 2000 mL crystalloid solution within 1h according to the vital signs and bleeding amount, with the infusion rate set to be faster than 20 mL/min. Thereafter, 1000 mL of colloid was infused to sustain the mean arterial pressure above 70 mmHg, after which the infusion rate was slowed down. A definitive operation was performed after resuscitation. Through the other channel, drugs for hemostasis and acidosis correction, hormones, and antibiotics were administered. Two channels identical to those in the control group were also established in the observation group. Through one channel, hydroxyethyl starch 40 resolved in 7.5% chloride sodium was rapidly infused within 10–15 min, while through the other channel, drugs that were used for hemostasis and to correct the acidosis, hormones, and antibiotics were administered. When the systolic pressure exceeded 70 mmHg, the infusion rate was lowered to keep the MAP between 61 and 70 mmHg, during which the infusion rate was dynamically adjusted according to the condition of patients with HTS. After infusion, effective hemostatic measures were immediately taken within 30–60 min. For any improvement in patients with HTS following the aforementioned treatment, rapid fluid resuscitation and blood transfusion were performed to rescue the state of ischemia and shock. Then, the infusion amount, bleeding amount, incidence rate of complications, and cure rate were recorded, and the hemodynamic indicators and coagulation indicators were identified and compared.

### Observation indicators

(1) During the treatment, the infusion amount, bleeding amount, incidence rates of complications (including the multi-organ dysfunction syndrome, acute respiratory distress syndrome, and acute renal failure), mortality rate, cure rate, and the total volume of infused fluid were observed and recorded.
(2) Before and after treatment, changes in the physiological indicators of patients in both groups, including temperature, Activated Partial Thromboplastin Time (APTT), Prothrombin Time (PT), and Plasma Fibrinogen (FIB) concentration were detected.

### Statistical analysis

Data analysis was performed using SPSS 20.0 software. GraphPad Prism 8.0 was used for plotting the data. Count data are presented as the rate or ratio, and the comparison of the cure rate between the two groups was performed using the Chi-Square test. Measurement data are represented as mean ± standard deviation, and the variation was testified using the *t-*test. A p-value of *<* 0.05 indicated statistical significance.

## Results

### General data of patients with HTS

The observation and control groups included 70 and 69 patients with HTS aged (27.8 ± 3.17) years and (28.18 ± 3.73) years, respectively. The two groups had no significant difference in age, cause of HTS, and grading of hemorrhagic shock, and were comparable (p > 0.05) (Table 1).

**Table 1:** General data of HTS patients (mean ± SD, years).

Group	n	Age	Cause of HTS			Grade of hemorrhagic shock		
			Fracture	Rupture of liver and spleen	Crush injury	II	III	Ⅳ
Observation group	70	27.8 ± 3.17	54	4	12	44	18	8
Control group	69	28.18 ± 3.73	45	5	19	45	15	9
p-value		0.874						

### Restrictive fluid resuscitation decreases the bleeding amount and incidence rate of complications in patients with HTS

By reviewing the treatment records of patients with HTS, it was found that there were significant differences in the bleeding amount, blood transfusion amount, and fluid volume between the control and observation groups (Table 2, p *<* 0.05). Meanwhile, patients in the observation group had a cure rate of 100%, which was significantly higher than 68.12% in the control group. Furthermore, no deaths were observed in both groups, while the incidence rates of MODS, ARDS, and ARF in the two groups had a significant difference (Table 3, p *<* 0.05).

**Table 2:** Bleeding amount, infusion volume and cure rate of HTS patients (mean ± SD, mL).

Group	n	Bleeding amount	Blood transfusion amount	Infusion volume	Cure rate (n)
Observation group	70	1336 ± 522.5	1432.6 ± 579.5	1566.1 ± 236.9	100% (70)
Control group	69	1803.4 ± 485.2	1991.6 ± 601.4	2589.2 ± 51.3	68.12 (47)
p-value		0.00122	0.00113	*<* 0.001	0.0056

**Table 3:** Incidence rate of complications of HTS patients.

Group	n	MODS (n)	ARDS (n)	AFR (n)	Incidence rate of complications (n)	Death rate
Observation group	70	7.14% (5)	4.29% (3)	1.42% (1)	12.9% (10)	0% (0)
Control group	69	26.09% (18)	21.74% (15)	13.04% (9)	60.87% (42)	0% (0)
p-value		0.036	0.034	0.029	< 0.001	1

MODS, Multiple Organ Dysfunction Syndrome; ARDS, Acute Respiratory Distress Syndrome; AFR, Acute Renal Failure.

### Restrictive fluid resuscitation promotes the restoration of hemodynamics in patients with HTS

**Fig. 1:**
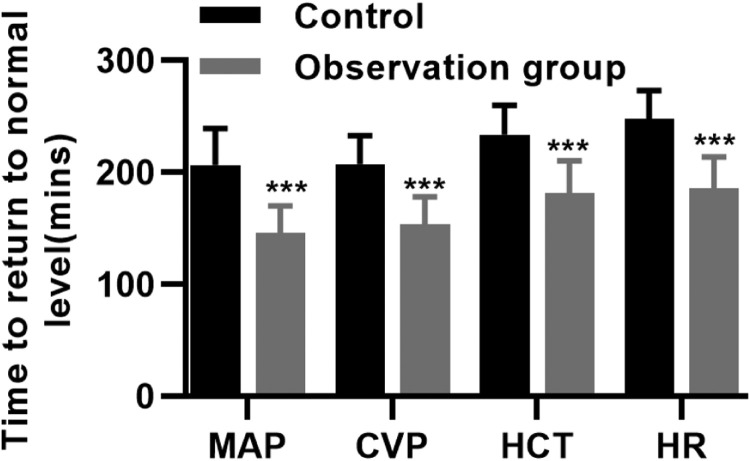
Time for MAP, CVP, HCT, and HR to return to the normal levels in the control and observation groups (*** p < 0.001).

**Fig. 2:**
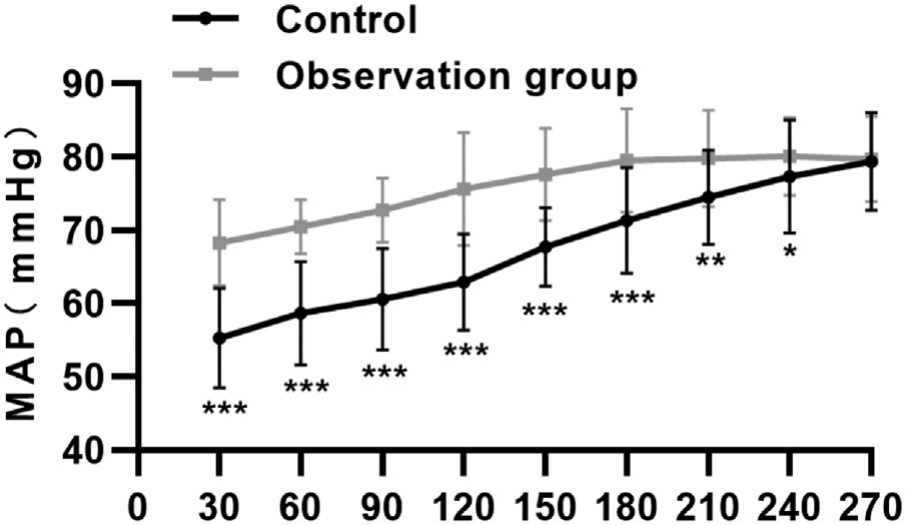
Curves describing the dynamic restoration of MAP to the normal level in patients in the control and observation groups (*p < 0.05, ** p < 0.01, *** p < 0.001).

After treatment, the restoration of hemodynamic indicators of patients in both groups was determined, and as a result, within 30 min and 2h after treatment, patients in the observation group had greater improvement in the MAP, CVP, HCT, and HR compared to those in the control group (Table 4, p *<* 0.05). Moreover, the time for the restoration of MAP, CVP, HCT, and HR to the normal levels and the dynamic course of MAP restoration were recorded, and it was found that patients in the control group required more time (Fig. 1, Fig. 2, p < 0.05).

**Table 4:** Hemodynamic indicators of HTS patients (mean ± SD).

Group	n	Time	MAP (mmHg)	CVP (mmHg)	HCT (%)	HR (beats/min)
Observation group	70	30 min	67.1 ± 6.1	7.8 ± 3.6	29.6 ± 4.7	91.4 ± 16.3
		2h	76.1 ± 6.7	11.4 ± 2.5	35.8 ± 6.3	78.6 ± 10.83
Control group	69	30 min	55.7 ± 7.2	5.3 ± 1.6	25.7 ± 5.5	100.2 ± 9.8
		2h	61.8 ± 7.1	7.6 ± 4.3	31.36 ± 6.8	84.6 ± 10.5
p-value		30 min	< 0.001	0.01	0.0243	0.005
		2h	0.0011	0.007	0.0176	0.008

### Restrictive fluid resuscitation accelerates coagulation function recovery in patients with HTS

Monitoring the changes in coagulation indicators can better reflect the adverse reactions of patients with HTS. In this study, the indicators of coagulation were continuously monitored. As a result, it was found that APTT, PT, and TT in the observation group had sharp decreases at 6h and 12h after treatment, while FIB increased significantly compared to that in the control group (Table 5, p < 0.05).

**Table 5:** Restoration of coagulation function of HTS patients (mean ± SD).

Group	n	Time	APTT (s)	PT (s)	TT (s)	FIB (g/L)
Observation group	70	6h	39.5 ± 7.1	14.7 ± 1.9	23.5 ± 3.2	2.4 ± 0.36
		12h	31.2 ± 5.6	12.1 ± 1.7	17.9 ± 3.5	3.11 ± 1.05
Control group	69	6h	48.5 ± 8.1	16.7 ± 5.1	23.8 ± 5.7	2.11 ± 0.82
		12h	35.2 ± 7.9	13.6 ± 3.8	21.8 ± 3.8	2.15 ± 0.57
p-value		6h	< 0.001	0.008	0.042	0.038
		12h	0.024	0.017	0.031	0.019

## Discussion

So far, HTS remains the leading cause of death and severe complications in patients in the emergency departments,^13^, ^14^, ^15^ and the current strategies for HTS treatment face significant challenges in spite of huge endeavors to improve patient prognosis. Although blood products remain the standard of care for hemorrhagic shock, they are a limited and perishable resource.^13^ Additionally, there is a growing concern that blood products have immunomodulatory effects that may negatively affect clinical outcomes.^13^ Therefore, developing resuscitation strategies that reduce the need for transfusion without increasing complications is crucial.

Fluid resuscitation is considered the main treatment for HTS.^16^ A large volume of fluid infusion, as soon as possible, can supplement the effective blood volume and maintain the blood pressure fluctuating in the normal range.^17^ However, the risk of bleeding may increase due to the difficulty in controlling active bleeding, migration of clots, reduction of blood coagulation factors, and blood dilution, concomitant with a significant increase in the incidence rate of complications in patients with HTS.^18^ Therefore, prior to controlling bleeding, the purpose of fluid resuscitation is to search for a balance between blood pressure and hemorrhage, so as to avoid the occurrence of complications such as MODS, ARDS, and ARF.

In clinical practice, restrictive fluid resuscitation has become a widely used method, which is superior to conventional fluid resuscitation.^19^ Restrictive fluid resuscitation is highly effective in increasing the plasma molarity in patients with HTS while being able to transfer the intercellular fluid to the vessels through the capillary wall, thereby increasing blood volume, increasing the returned blood volume and rapidly augmenting the arterial pressure, and stabling the circulation system.^20^,^21^ Moreover, restrictive fluid resuscitation can optimize the blood flow and reduce the adhesion of blood cells to the vascular wall to decrease peripheral vascular resistance and improve microcirculation in patients with HTS.^22^,^23^

In this study, restrictive fluid resuscitation was used for the treatment of HTS, and the results showed that in comparison with the control group, patients in the observation group had a sharp decrease in the total volume of blood transfusion and infusion volume, while the incidence rates of MODS, ARDS, and ARF showed that patients in the observation group had a lower incidence rate of complications and a more rapid restoration of hemodynamic indicators. Hence, restrictive fluid resuscitation is highly effective for treating HTS by reducing persistent hemorrhage and improving the hemodynamic indicators, oxygen-carrying capacity of tissue, and coagulation function compared to conventional fluid resuscitation strategies.

After reviewing the treatment records of patients with HTS, it was found that patients in the observation group had a more rapid recovery of coagulation function than those in the control group, and the coagulation function of patients in the observation group was much superior to that of those in the control group, suggesting that restrictive fluid resuscitation was more efficient in improving the coagulation function. In patients with HTS, the persistent depletion of blood contributes to the loss and consumption of platelet, FIB, and other coagulative factors in the blood, while fluid resuscitation is a strategy for treating patients with HTS by the infusion of large amounts of fluid, which, however, results in the decrease in the coagulative factors due to the dilution. Besides, continuous blood loss causes a decrease in temperature, hypocalcemia, anemia, acidosis, and hyperfibrinolysis, which further leads to coagulation dysfunction, or even diffusive intravascular coagulation, thus aggravating bleeding and forming a vicious cycle. In this study, the authors believed that restrictive fluid resuscitation using the hydroxyethyl starch in chloride sodium to substitute the plasma can rapidly increase the plasma osmotic pressure to facilitate the return of tissue fluid to enlarge the blood volume, dilute the blood, depolymerize the blood cells, reduce the adhesion of blood cells, and increase the negative charge of the membrane, thereby improving the microcirculation. The improvement of microcirculation can further reduce the factors that interfere with coagulation and restore coagulation function, while the application of restrictive fluid resuscitation can reduce the volume of fluid infusion to decrease the dilution of coagulation factors and improve the coagulation function. However, this study has certain limitations. Due to the small number of patients admitted to the hospital, the sample size is small. Besides, patients with HTS are a special group, and the treatment method of restrictive fluid resuscitation is relatively simple, different treatment methods are needed to solve the complicated complications encountered in the treatment process. In addition, restrictive fluid resuscitation in patients with HTS has not been extensively studied in the past few years. With the development of medical technology, further studies on restrictive fluid resuscitation should involve a large number of clinical patient trials.

In conclusion, restrictive fluid resuscitation can effectively improve coagulation function and reduce the incidence rate of complications in patients with HTS, which is conducive to the restoration of temperature and hemodynamic indicators and reduction of bleeding amount. Additionally, it can regulate the MAP between 61 and 70 mmHg as the balance point between the blood pressure and bleeding event. Furthermore, it can reduce the amount of bleeding and infusion volume and increase the cure rate. Therefore, restrictive fluid resuscitation has a promising value in the treatment of HTS, which is worthy of promotion in clinical practice.

## CRediT authorship contribution statement

**Junfen Zheng:** Conceptualization, Data curation, Formal analysis, Methodology. **Jinqiang Zhu:** Conceptualization, Data curation, Formal analysis. **Liexiang Cao:** Data curation, Formal analysis, Software. **Meiping Dong:** Data curation, Formal analysis, Supervision. **Yi Mao:** Data curation, Formal analysis, Validation. **Zhiwei Zhao:** Data curation, Formal analysis, Validation. **Yao Liu:** Data curation, Formal analysis, Methodology, Writing – original draft, Writing – review & editing.

## Declaration of Competing Interest

The authors declare no conflicts of interest.
